# Controlled acceleration of GeV electron beams in an all-optical plasma waveguide

**DOI:** 10.1038/s41377-022-00862-0

**Published:** 2022-06-14

**Authors:** Kosta Oubrerie, Adrien Leblanc, Olena Kononenko, Ronan Lahaye, Igor A. Andriyash, Julien Gautier, Jean-Philippe Goddet, Lorenzo Martelli, Amar Tafzi, Kim Ta Phuoc, Slava Smartsev, Cédric Thaury

**Affiliations:** 1 LOA, CNRS, Ecole Polytechnique, ENSTA Paris, Institut Polytechnique de Paris, 181 Chemin de la Hunière et des Joncherettes, 91120 Palaiseau, France; 2 Department of Physics of Complex Systems, Weizmann Institute of Science, Rehovot, 76100 Israel

**Keywords:** Plasma-based accelerators, Laser-produced plasmas

## Abstract

Laser-plasma accelerators (LPAs) produce electric fields of the order of 100 GV m^−1^, more than 1000 times larger than those produced by radio-frequency accelerators. These uniquely strong fields make LPAs a promising path to generate electron beams beyond the TeV, an important goal in high-energy physics. Yet, large electric fields are of little benefit if they are not maintained over a long distance. It is therefore of the utmost importance to guide the ultra-intense laser pulse that drives the accelerator. Reaching very high energies is equally useless if the properties of the electron beam change completely from shot to shot, due to the intrinsic lack of stability of the injection process. State-of-the-art laser-plasma accelerators can already address guiding and control challenges separately by tweaking the plasma structures. However, the production of beams that are simultaneously high quality and high energy has yet to be demonstrated. This paper presents a novel experiment, coupling laser-plasma waveguides and controlled injection techniques, facilitating the reliable and efficient acceleration of high-quality electron beams up to 1.1 GeV, from a 50 TW-class laser.

## Abstract (web summary)

Coupling of laser-plasma waveguides and controlled injection techniques produce high quality GeV electron beams for the first time.

## Graphical abstract

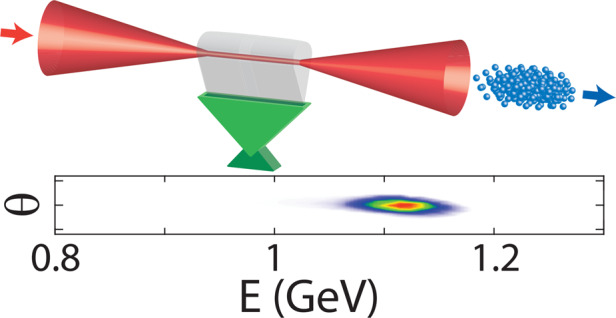

## Introduction

Particle accelerators are indispensable tools for science and technology that allow for the scrutiny, manipulation, and even creation of matter and light. Conventional accelerators provide energy to charged particles by exciting radio-frequency waves in metallic cavities. The magnitude of the accelerating field is ultimately limited by the material breakdown. Since the 1960s^1^, scientists have considered plasma-based accelerators as an alternative. Plasma, being already ionized, does not break down, and thus can theoretically sustain arbitrarily strong electric fields. Today’s plasma accelerators, driven by high-power lasers, have evolved from early concepts^2^ to devices, which are able to operate accelerating fields over three orders of magnitude higher than radio-frequency cavities. However, despite the tremendous progress made by laser-plasma accelerators from early demonstrations^3–9^, the quality of the delivered electron bunches still does not match that of conventional machines. In particular, it is challenging for laser-plasma accelerators to accurately control the particles phase in the accelerating field, and to keep the beam in a strong field over long distances.

The basic recipe for building a laser-plasma accelerator is straightforward: it consists of focusing an ultra-high intensity laser pulse in a gas which is turned into a plasma. As the laser propagates, it expels all plasma electrons out of its way, and thus generates in its wake a positively charged cavity^10^. The fields in this cavity, also known as wakefields, reach values of the order of 100 GV m^−1^. The whole picture gets more complicated when it comes to injecting electrons into the wakefield. In almost all laser-plasma accelerators, the injection is achieved through the capture of plasma electrons, which are provided with enough energy to satisfy the trapping condition (i.e., so that the velocity of the electrons exceeds the cavity velocity)^10^. Several schemes have been developed to control the injection; they include optical injection^9^, injection in a steep density gradient^11^, localized ionization injection^12,13^, or a combination of these methods^14^. They allow for the production of electron bunches with a relative energy spread of the order of one percent for 200–300 MeV electrons^15,16^, normalized transverse emittance as small as 0.1 mm.mrad^17^, and a stability in charge and energy of a few percent-limited by that of the laser^16^. As such, the beam quality has been proven sufficient to drive a compact X-ray free-electron laser^18^, thus approaching that of conventional devices. Further advances in laser stability and repetition rate^19^ should bring laser-plasma accelerators into real competition with them in the sub-GeV range.

At higher energies more problems arise, in particular the difficulty to sustain the electric field over a long distance. Achieving the required guiding of the laser pulse while preserving a high beam quality, has emerged as one of the most important challenges for laser-plasma acceleration. A breakthrough was achieved in 2008 with the acceleration of an electron beam up to an energy of 1 GeV, in a plasma waveguide^8^. Such a waveguide consists of a plasma channel whose electron density *n*_e_ decreases toward the optical axis. The refractive index of plasma varies inversely with the density, therefore the plasma channel acts as a graded-index optical fiber^20^, able to guide an intense laser pulse. In the context of laser-plasma acceleration, the only technique demonstrated so far to generate a plasma waveguide is the capillary discharge^21^. This technique uses a device, which consists of a gas-filled capillary to the ends of which is applied a high voltage pulse. The resulting discharge ionizes the gas along the capillary axis and produces a hot plasma which then radially expands for a few nanoseconds, generating the waveguide. This technique was successfully used to break several energy records, from 1 GeV with a 1.6 J laser pulse^8^ and up to 7.8 GeV with a 31 J pulse^22^. However, the capillary discharge has a few drawbacks. First, at low plasma density, the channel is not deep enough to effectively guide the laser, meaning that a significant amount of energy can reach the walls of the capillary and damage it. As a consequence, in order to accelerate electrons above 4 GeV, it is necessary to use an additional laser pulse to further deepen the plasma channel^23^. Moreover, the fact that any leakage of energy out of the guide can damage the capillary raises doubts about the possibility of using this device intensively at high repetition rates and high laser energies. Finally, this device has never been combined with a controlled injection technique. Thus, it often results into the acceleration of unstable and poor-quality electron beams.

These shortcomings have led to the search for new guiding methods, and optically-made plasma channels have emerged as promising alternatives. Such concept of plasma guiding is well known, and was demonstrated in 1993 using a 100 picosecond laser pulse. In this scheme, the laser is focused to a line by an axicon lens in order to produce a plasma column from an Ar-gas, through collisional ionization^24^. The radial expansion of the plasma then leads to the formation of a waveguide, similar to that created by a capillary discharge. This idea was recently revived by the use of femtosecond lasers which produce the plasma through optical field ionization^25^. In contrast to collisional ionization which is not effective at the low densities required for high-energy plasma accelerators (*n*_e_ ≲ 10^18 ^cm^−3^), the efficiency of field ionization does not depend on the gas density. Different implementations of this laser-generated waveguide have successfully allowed for cm-scale guiding of laser pulses of relativistic intensity (*I ≳* 10^19 ^W cm^−2^)^26^ and for meter length guiding of weaker laser pulses at densities as low as 5 × 10^16 ^cm^−3^^27,28^^,^. It is therefore perfectly suited for laser-plasma acceleration, which requires maintaining the laser focus at relativistic intensities in a plasma with densities 10^16^ ≲ *n*_e_ ≲ 10^18 ^cm^−3^. It is also particularly fit to be used with controlled injection as it does not impose significant constraints on the shaping of the plasma density profile, the employment of gas mixtures or multiple laser beams.

In the experiment presented here, we use a laser-generated plasma channel to guide an intense laser pulse over 15 mm and show the efficient generation of 1.1 GeV electron beams, from a 1.7 J / 30 fs laser pulse. The versatility of this technique is demonstrated using two different injection methods, ionization injection and density transition injection, leading either to broadband spectra with a high total charge, or electron beams with narrow spectra and a higher charge density at the GeV level. This control of the injection into a plasma guide is a major advance toward the realization of a true plasma accelerator at very high energy.

## Results

**Fig. 1:**
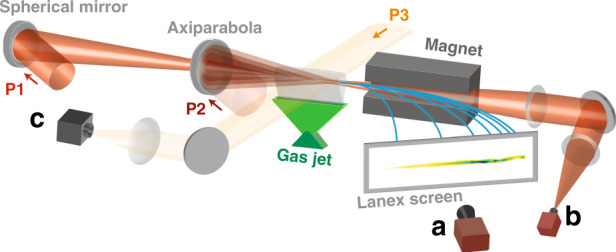
Schematic view of the experiment and main diagnostics. The laser is split into three separate beams: P1 - the acceleration driver, P2 - the pulse that generates the waveguide, and P3 - the probe beam. The two main beams, P1 and P2, are focused in a 15 mm long rectangular gas jet. The generation beam, P2, is focused in the target, 2 ns before P1, by an f/4 axiparabola. The main beam, P1, is focused by an f/18 spherical mirror. It accelerates electron bunches whose energy is then analyzed with a spectrometer consisting of a dipole magnet, a LANEX scintillating screen and a 16 bits camera **a**. It is eventually attenuated to image its focal spot after interaction (**b**). The probe beam, P3, crosses the plasma transversely, just after P1 (**c**). It is then sent to a wavefront sensor to measure the transverse density profile

**Fig. 2:**
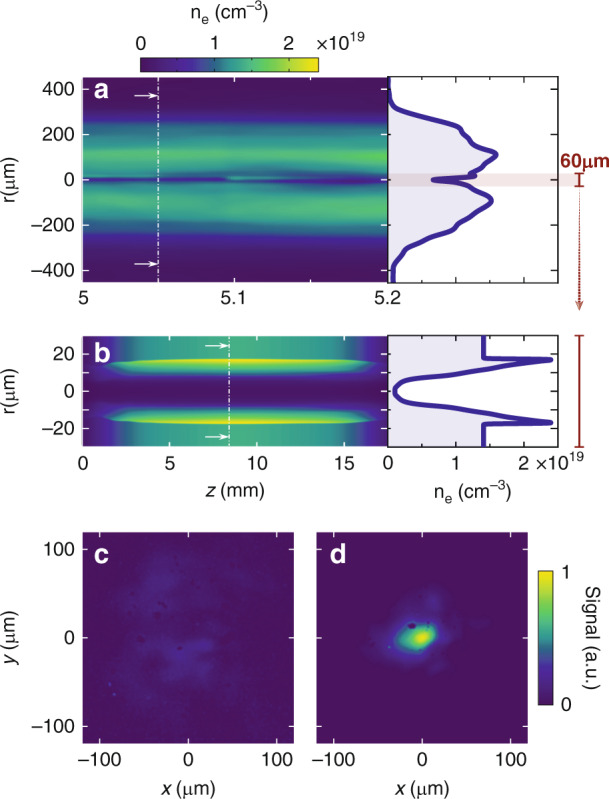
Plasma channel and guiding. **a** Measured density profile. The density in the middle of the waveguide is overestimated because of limited resolution. **b** Simulated plasma channel (see Methods). **c**, **d**, Laser focal spot of P1, measured after attenuation of the full energy beam at the exit of the plasma, without (**c**) and with (**d**) guiding

The experimental setup is shown in Fig. 1. A 1.5 mJ, 30 fs laser pulse, P2, is focused by an axiparabola^26^ into a supersonic gas jet, generating a 15 mm long plasma filament (see Fig. S3 in Supplementary Information). The plasma then expands radially and after 2 ns a plasma waveguide is formed^25^. Figure 2a shows a typical density profile measured after the formation of the waveguide. The central part of this profile was simulated using a hydrodynamic code (see Methods). From these simulations, we estimate that the guide has a full diameter of ~30 µm, and an axial density of (1.4 ± 0.3) × 10^18 ^cm^−3^ (see Fig. 2b). The main beam, P1, is focused at the entrance of this waveguide, and is effectively guided over 15 mm. This guiding is illustrated in Fig. 2c, d by focal spots measured at the exit of the plasma, without and with the waveguide respectively (*i.e*. without and with the P2 laser pulse). More details on the plasma waveguide can be found in Supplementary Information.

**Fig. 3:**
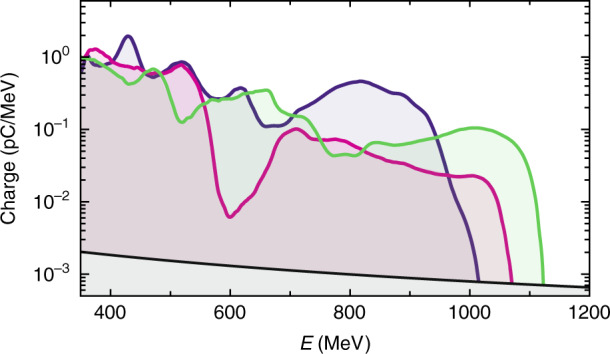
Acceleration of electron beams with broad energy distributions. Three consecutive electron spectra obtained using ionization injection. The black line indicates the detection threshold.

Initially, the setup was used with a gas mixture target so as to trap electrons into the accelerating field through ionization injection^29^. This easily implementable injection mechanism generally yields electron beams with a broad energy distribution and a high total charge. Three consecutive spectra obtained in this regime are displayed in Fig. 3. As expected, spectra are quasi-continuous with a maximum energy of about 1.1 GeV. The uncertainty on the energy at 1 GeV is 44.7 MeV (see Methods and Supplementary Information). The results are consistent with Lu’s model^30^, which estimates the energy gain and acceleration length in an ideal case, and predicts an acceleration length of 15 ± 3 mm and an energy gain of 1.3 ± 0.3 GeV for an electron density *n*_e_ = (1.4 ± 0.3) × 10^18 ^cm^−3^. The total charge above 350 MeV exceeds 50 pC, meaning that about 2.2% of the laser energy in the laser focal spot was transferred to electrons above 350 MeV. Around 70% of the shots show electrons above 600 MeV. The absence of electrons above this energy for some shots is correlated with a poor guiding of the laser energy, and attributed to pointing fluctuations between the two laser beams (see Methods and Supplementary Information), which prevents an effective coupling of the main beam into the waveguide. This issue could be solved by using dynamic correction of the laser pointing^31,32^.

**Fig. 4:**
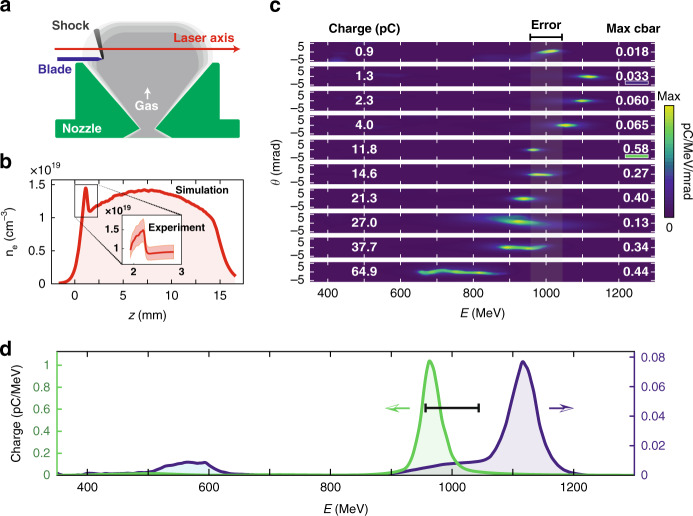
Acceleration of high-quality electron beams with density transition injection. **a** Schematic view of the gas target. **b** Density profile obtained from a fluid simulation for our experimental parameters and measured density profile in the shock region (inset). **c** Ten angularly resolved electron spectra sorted by charge (charge in the peak for *d*^2^*N/dEdθ* > 0.1(*d*^2^*N/dEdθ)*_max_). **d** Two examples of angularly-integrated spectra corresponding to spectra marked by blue and green lines in (**c**). The black segment indicates the uncertainty on the energy due to fluctuations of beam pointing

This first experiment demonstrates that an intense laser can efficiently drive a wakefield and then trap and accelerate an electron beam in a laser-generated plasma waveguide. One of the main advantages of this approach is that the use of a free gas flow target allows plasma density to be shaped without affecting the guiding efficiency. Therefore, in the second experiment we have combined the developed waveguide with density transition injection to control the trapping position of electrons into the accelerator and thus the final beam energy. This injection strategy requires a sharp density down-ramp in the region of the target where the injection is desired. This down-ramp induces a sudden increase in the length of the accelerator cavity which leads to the injection of electrons from the back of the cavity into the accelerating field^33^. In practice, this density transition is obtained by obstructing the gas flow on one side of the nozzle exit to generate a hydrodynamic shock, as first demonstrated in Ref. ^34^ and illustrated in Fig. 4a, b.

The narrowing of the energy distribution, allowed by the control of the injection, is illustrated in Fig. 4c by a set of 10 spectra, sorted by increasing charge. These spectra were selected from a series of 14 consecutive shots, excluding four shots with a negligible charge which were attributed to pointing fluctuations and the resulting misalignment of the waveguide. As can be seen, a well-peaked spectrum is obtained for all successful shots. The conversion efficiency from the laser to the high energy peak is 1% for 1 GeV beams and can be as high as 6% for the most loaded ones, comparable to the most efficient wakefield accelerators^35,36^. Figure 4d shows the angularly integrated spectra of two of these shots. The observed energy spread is 4.5% Full Width at Half Maximum (FWHM) for the beam in the series with the highest energy (blue curve) and 3.6% FWHM for the one with the lowest energy spread (green curve). These spreads are biased by the beam divergence; deconvolution from the divergence measured in the vertical direction leads to energy spreads of 3.7% FWHM for the blue curve and 2% for the green one FWHM. Figure 4c also exhibits a clear correlation between the charge of the peak and its energy. This can be attributed to beam loading, or in other words, to the screening of the accelerating field by the beam itself. A precise control of this loading can allow for the flattening of the accelerating field so that the entire electron beam experiences the same field. Such fine control was shown to produce energy spread as low as 2 MeV FWHM with 200–300 MeV-class laser-plasma accelerators^15,16^.

## Discussion

In summary, we demonstrated for the first time electron acceleration in a laser-generated plasma waveguide, and showed the acceleration of electron beams up to the GeV range using a 50 TW-class laser. We showed that such waveguides can be combined with a controlled injection technique to obtain high-quality, GeV scale electron beams. In this proof of concept experiment, the energy spread was measured to be below 4% FWHM for the best shots. The coupling efficiency from the main laser beam into the waveguide varies shot-to-shot because of pointing fluctuations. This leads to significant variations of the trapped charge and to about 30% of shots failing to capture electrons. To the best of our knowledge, this is the lowest percentage of missed shots reported so far for electron acceleration in a plasma waveguide (for both laser-generated waveguides and capillary discharges). Implementation of active or passive pointing stabilization should significantly reduce this number.

Optically generated plasma channels have already demonstrated their ability to efficiently guide laser pulses over meter distances, using less than 0.1 J m^−1^ to form the guide^27,28^. Thanks to optical field ionization and its immunity to laser damage, our approach should therefore be scalable to the most powerful lasers and to higher repetition rates. The scheme promises to actualize multi-GeV beams for a free-electron-laser^18^ or a collider injector^37^. The achieved energy is ultimately limited by dephasing, i.e. the electron beam going faster than the laser pulse in the plasma and eventually exiting the accelerating region of the wakefield. This effect can be mitigated by using a rising density profile to increase the energy by up to a factor of two^38–40^. As the efficiency of optical field ionization does not depend on the plasma density, the longitudinal density profile in the waveguide could be optimized to further boost the energy.

## Materials and methods

### Laser

The experiment was conducted at Laboratoire d’Optique Appliquée on a Ti:Sapphire laser system delivering 70 TW on target at a central wavelength of 810 nm, divided in three 30 fs pulses. One arm, P1, containing an energy of 1.7 J is used for electron acceleration. It was focused into the gas jet with a 1.5-m-focal-length spherical mirror, to a focal spot size of 13.5 µm (FWHM) to reach an intensity of ≈ 2 × 10^19 ^W cm^−2^ (normalized vector potential *a*_0_ ≈ 3). The encircled energy within the first dark ring was estimated to be 60%. The pointing stability is about 3 µrad and 2 µrad Root Mean Square in the horizontal and vertical planes respectively, corresponding to lateral shifts in the focal plane of 4.4 µm and 3.1 µm. The second arm, P2, is used for generating the plasma waveguide. It is attenuated to an energy of 1.46 mJ and focused by a holed axiparabola with a nominal focal length of *f*_0_ = 200 mm and a focal depth of *δ* = 30 mm, 2 ns before the main pulse. The focal line of the axiparabola is defined by *f* (*r*) = *f*_0_ + 1*/a* ln(*r/R* × e^*aδ*^) with *a* = 1*/δ* ln(*R/r*_*hole*_) where *r*, *r*_*hole*_ = 8 mm, *R* = 38.1 mm are respectively the radial coordinate, the radius of the hole in the center of the axiparabola, the axiparabola radius. The peak intensity at focus is ≈ 5 × 10^15 ^W cm^−2^, and the focal spot diameter at first zero decreases from 15.5 µm down to 12 µm at the end of the focal line. The focal spots of both beams were optimized using two independent deformable mirrors. A third low-energy beam, P3, was used to probe the plasma.

### Target

For the ionization injection experiment, the gas was a mixture of 99% Hydrogen with 1% of Nitrogen. The backing pressure was 40 bars, and the measured electron density is about 1.4 × 10^19 ^cm^−3^. For the density-transition injection experiment, a pure hydrogen gas was used. The backing pressure was 40 bars, and the measured electron density in the plateau region of the unperturbed plasma of approximately 1.4 × 10^19 ^cm^−3^. In both cases, the density was measured at each shot, using a probe beam to transversely image the target. The phase introduced by the plasma was measured using a wavefront sensor (160 × 120 px^2^ resolution), and the corresponding density profile reconstructed, assuming cylindrical symmetry around the laser axis and using Abel inversion. The wavefront sensor was mounted on a translation stage to be able to reconstruct the full target. In addition, the gas flow produced by the nozzle was simulated using the commercial code Ansys FLUENT.

### Simulation of the plasma channel formation

For more details on the channel features, its creation was simulated numerically. The field of the axiparabola beam was computed at multiple positions along the focal line, and the electron temperature and ionization state were calculated, at each position, considering collisionless above-threshold ionization heating. Finally, the plasma channel radial expansion was modeled using a plasma hydrodynamic simulation considering the obtained electron temperature, ionization state and gas density. Note that the plasma expansion simulation does not provide exactly the same information than the experimental data in Fig. 2a. While we measured the density of the plasma electrons in the experiment, the simulation provides the full electron density, including neutrals, ions and free electrons. More precisely, we plot in Fig. 2b the distribution of *n*_p_ = *ρ/A*_H_, where *ρ* is the mass density of the media, and *A*_H_ ≃ 1 is the atomic weight of Hydrogen. This leads to a constant density outside the channel in Fig. 2b, while Fig. 2c shows the radial profile of the plasma.

We have considered an ideal axiparabola with the same parameters as the experimental design: a 200 mm focal distance, a 30 mm-long focal line, a 38.1 mm radius and a central hole of *R*_hole_ = 8 mm. The laser beam was initialized on the axiparabola surface with a top-hat spot of *R*_laser_ = 27.5 mm, and a duration of 28 fs. The laser energy was considered to be 0.5 mJ in order to account for the non-ideal features of the laser beam and the axiparabola. The optical propagation was modeled using the Axiprop library^41^. More details on such simulations can be found in ref. ^42^. At the different radii and positions along the focal line, the ionization was calculated via ADK model, and the resulting electron energies were calculated from the laser field potential at the moment of ionization^43^. This simplified approach was cross-checked against a more complete particle-in-cell modeling and has shown a good agreement.

For the simulation of plasma expansion, we used the parallel multidimensional Eulerian hydrocode FRONT^44^. This code uses Riemann solvers for hyperbolic equations and has a number of physical models implemented as modules. Here, we considered separately electron and ion fluids, and computed the electron thermal conductivity and atomic ionization kinetics. More information on the plasma channel formation simulations is provided in Supplementary Information.

### Electron spectrometer

Electrons are dispersed by a 0.85 T, 400 × 80 mm^2^ U magnet on a 365 mm-long Kodak Lanex Regular screen that is imaged by a 16-bit CCD Andor camera. An interference filter at 546 nm is placed in front of the camera to minimize parasite light. Absolutely calibrated radioactive Tritium light sources are attached to the scintillating screen to provide charge calibrations^45^ with 28% precision. A 500 µm wide slit placed in front of the magnet limits the acceptance angle within the dispersion plane down to 2.5 mrad, thus resulting in a statistical error on the beam energy of 4.4% at 1 GeV. Instead, the systematic error is mainly related to the imaging resolution within the dispersion plane and to the uncertainties on the relative positions of the gas jet, dipole and Lanex screen. This is estimated to be 0.81% at 1 GeV. Finally, the transverse resolution of the imaging system limits the resolution of the divergence measurements at 0.4 mrad. More information on the spectrometer calibration, as well as on the systematic and statistical errors is provided in Supplementary Information.

## Supplementary information

Supplementary Material
