# Supplementary material for: Controlled acceleration of GeV electron beams in an all-optical plasma waveguide
Source: Light Sci Appl. 2022 Jun 14;11:180. doi: 10.1038/s41377-022-00862-0 (PMC9198076; doi:10.1038/s41377-022-00862-0)
Supplement: Supplementary file 1 — Supplementary Material [file 41377_2022_862_MOESM1_ESM.pdf]

Supplementary Information for
Controlled acceleration of GeV electron beams in an all-optical plasma waveguide

Kosta Oubrerie[1], Adrien Leblanc[1], Olena Kononenko[1], Ronan Lahaye[1], Igor A. Andriyash[1], Julien Gautier[1], Jean-Philippe Goddet[1], Lorenzo Martelli[1], Amar Tafzi[1], Kim Ta Phuoc[1], Slava Smartsev[1,2], and Cedric Thaury[1,*]

[1]LOA, CNRS, École Polytechnique, ENSTA Paris, Institut Polytechnique de Paris, 181 Chemin de la Hunière et des Joncherettes, 91120 Palaiseau, France
[2]Department of Physics of Complex Systems, Weizmann Institute of Science, Rehovot 76100, Israel

This supplementary information provides a description of the electron spectrometer and discusses the sources of statistical and systematic errors on the electron energy. It then presents additional data on the formation and optimization of the optical plasma waveguide, as well as correlations between the guiding efficiency and the electron beam features.

1 Electron spectrometer

Experimental Setup. The electron spectrometer consists of a dipole magnet which disperses the electron bunch within the plane perpendicular to the poles towards a scintillating screen (Fig. S1a). Electrons from the laser-plasma accelerator propagate 204 ± 2 mm in vacuum before reaching the magnet entrance. A collimating slit is placed in front of the dipole, at 200 ± 2 mm from the target. This aperture is made of two 1 mm thick inox steel plates that are separated by 500 μm distance. The gap of the slit is perpendicular to the magnet dispersion plane, in order to leave unaffected the vertical divergence of the electron beam. The slit enables the reduction of the lateral acceptance angle down to 2.5 mrad. The light emitted by the scintillating screen is then collected by a 16 bit CCD IDus Andor camera with a 546 nm (10 nm FWHM) Alluxa interferential filter in front of the objective.

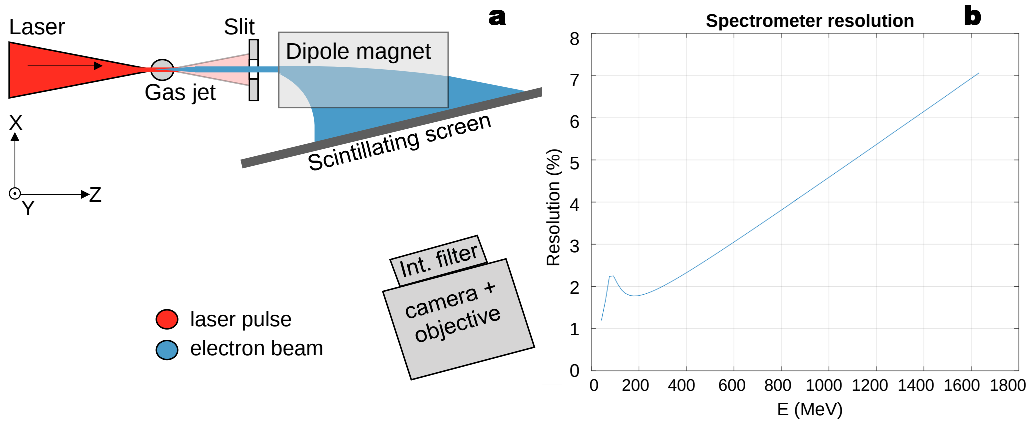

Figure S1: (a) Schematic of the electron spectrometer. The laser pulse is focused on a gas target that is placed 204 mm before the entrance of the dipole. In front of the dipole, a vertical slit is inserted to limit the acceptance angle of the electron beam to 2.5 mrad. Electrons from the target propagate through the magnetic field towards the scintillating screen, that is imaged by a CCD camera. (b) Resolution of the electron spectrometer as a function of the beam energy for an acceptance angle of 2.5 mrad.

Magnetic Field. In this experiment, we used a 40 cm long, 8 cm wide U-shape dipole with 2 cm gap between the poles, providing 0.85 T transverse magnetic field B_y. The 2D distribution of B_y was measured using a Hall probe (Fig. S2a) with a 1 mm spatial resolution.

Around the edges of the magnet the fringe field is inhomogeneous, and it acquires B_z and B_x components (see Fig. S2b). According to the Hall probe measurements shown in Fig. S2c, the longitudinal field B_z appears negligible except in a 6 cm region at the entrance/exit edges. Close to the dipole edges this field reaches a maximum value of 24 mT on axis. The component B_x is also noticeable only at the dipole edge where it increases with the distance to the axis. For a 1.25 mrad transverse angle it reaches a maximum value of 75 mT.

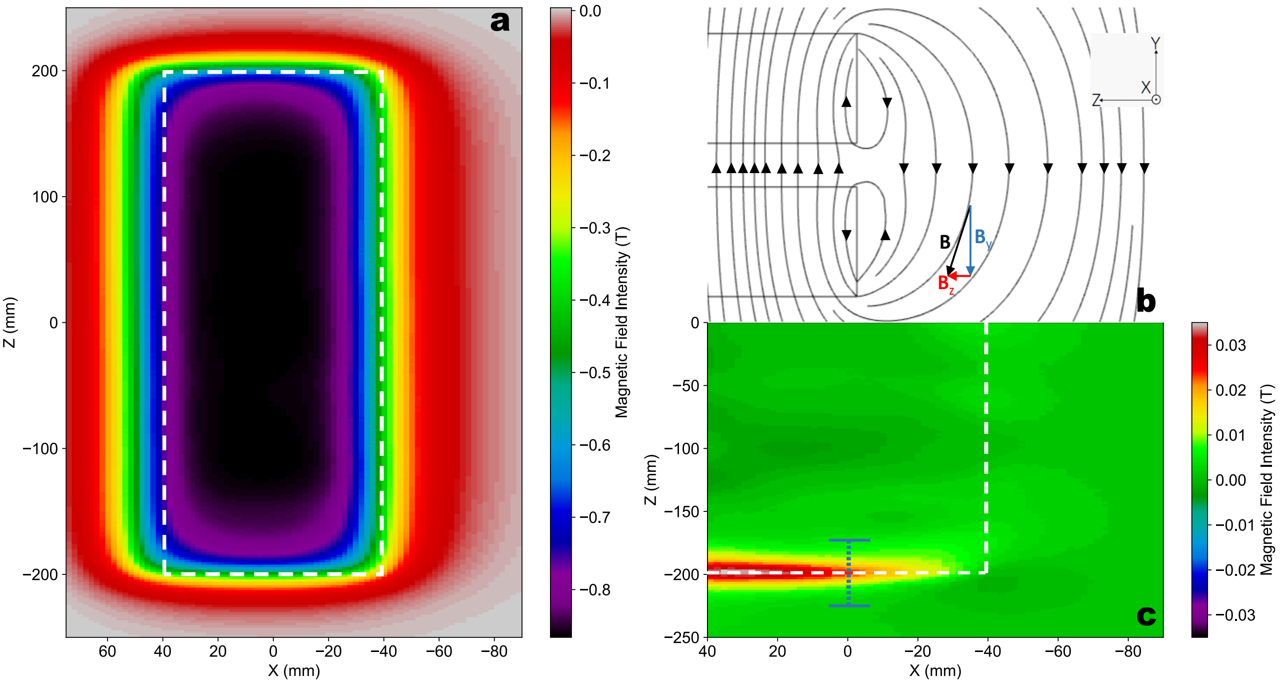

Figure S2: Magnetic field measurements. (a) Transverse component B_y corresponding to the dispersion of the electrons towards the scintillating screen. The white dotted profile corresponds to the magnets position. (b) Schematic showing the side view of the dipole entrance, B_y and B_z components of the magnetic field. (c) Longitudinal component B_z. The blue dotted line is indicating the 6 cm region of maximum intensity.

Detector screen. The particles are detected using a Lanex regular Carestream scintillator, oriented at $58.4° \pm 1°$ with respect to the laser axis. This scintillator is made of three layers: a plastic support layer, a sensitive layer with Gadolinium oxysulfide (Gd_2O_2S, also called gadox) and a protective thin plastic layer, with a total thickness of 380 μm. The Gadox emits light with a spectrum spanning from 382 to 622 nm, and a peak at 545 nm. The imaging resolution of the Lanex surface in the dispersion plane of 0.313 mm/pixel results in an energy resolution of 0.8% at 1 GeV. The transverse resolution of 0.2667 mm/pixel defines the resolution of the divergence measurements of 0.4 mrad.

Calibration. For the charge calibration, we have used two 5×2 mm^2 radioactive tritium capsules with a luminescent phosphor layer and an initial activity of 0.76 GBq. These sources were absolutely calibrated to the emission efficiency of Carestream Regular scintillating screen at the conventional ELBE accelerator, where absolute fluorescence efficiency was found to be $(3.1 \pm 0.6) \times 10^9$ ph sr^{-1} pC^{-1}. The capsules were attached close to the area of the Lanex screen where the luminescence from electrons was observed. This allowed to account for different aspects of imaging conditions that are not sensitive spectral-wise, as for instance, the numerical aperture of the camera, the signal attenuation by neutral density filters and the quantum efficiency of the camera. The interference filter in front of the camera was calibrated in a separate experiment by measuring an attenuation for a large series of stable electron spectra.

Measurement interpretation. The electron trajectories in the magnetic field define the position at which the electrons are hitting the detector plane. The energy is estimated from the detection position on the screen, and the charge is deduced from the intensity of the emitted light. For the spectrum measurement interpretation, the electron trajectories from the source to the scintillating screen were computed numerically using a 2D particle tracking code and the measured magnetic field.

Measurement error. The electrons are injected in the laser-plasma accelerator with a microradian pointing precision, and the source position jitter is negligible compared to the 204 mm travel distance to the spectrometer. Before the dipole magnet, the slit selects the lateral angles with an acceptance of 2.5 mrad. The slit has two functions: it restricts the electron beam pointing – the source of the statistical error, and reduces the uncertainty on the beam propagation axis – the main source of the systematic error. The latter corresponds to a transverse momentum of the beam, and results in an error on the measured energy, whose magnitude depends on the beam energy. Figure S1b illustrates how the relative error varies with the beam energy for an acceptance angle of 2.5 mrad.

The inhomogeneities of the magnetic fringe field may also cause deviations of the electrons tracks, e.g. produce an energy dependent focusing in the vertical direction for the off-axis particles. The resulting error on the divergence measurement is given by $\delta\theta \simeq ecB_{\rm int}/E$, where $\delta\theta$ is the deflection angle due to B_x, e and c are the electron charge and speed of light constants, $B_{\rm int}$ is the integrated magnetic field, and E the electron energy. According to Hall probe measurements, $B_{\rm int} \approx 7.5 \times 10^{-4}$ T m so the deflection angle does not exceeds 0.5 mrad for 1 GeV beams.

At the exit of the magnet, the longitudinal component of the magnetic field can introduce an angular shift in the trajectory of the electrons. Using the Hall probe measurement shown in Fig. S2c, and using the same formula used to estimate the defocusing effect, this deflection for a 1 GeV beam crossing the 6 cm region highlighted on the magnetic map amounts to a angle of 0.17 mrad. This angular shift corresponds to a shift of 10 μm of the position on the detector, which is lower than the imaging resolution of 0.8%. This deflection effect at the exit of the magnet is thus negligible for a 1 GeV electron beam.

The uncertainty on the Lanex angle and on the distance between the gas jet and the magnet leads to an error of 0.063% and 0.046% for 1 GeV electrons respectively. By taking into account all the aforementioned uncertainties, we obtain a total error of 4.47%.

The charge calibration is provided by the tritium capsule enclosed to the detector screen. Assuming an error of 20% on the count of the number of photons from the capsule due to a potential degradation of the luminescent material, in addition to the 19% error on the source calibration performed at ELBE, the total error on the charge measurement is estimated to be 28%.

2 Optical plasma waveguide

Longitudinal density profile. The gas target was produced from a 3D-printed, rectangular nozzle. The exit aperture was 15×0.5 mm^2, and the nozzle throat 4π mm^2 with a 22.5 mm transition between these two sections. The phase introduced by the plasma was measured using a wavefront sensor (160×120 px^2 resolution), and the corresponding density profile reconstructed, assuming cylindrical symmetry around the laser axis and using Abel inversion. The wavefront sensor allows to measure the density over a length of approximately 0.95 mm. It was mounted on a translation stage to be able to reconstruct the full target. In addition, the gas flow produced by the nozzle was simulated using the commercial code Ansys FLUENT. The measured longitudinal density profile is compared with the simulation in Fig. S3, for a backing pressure of 40 bars.

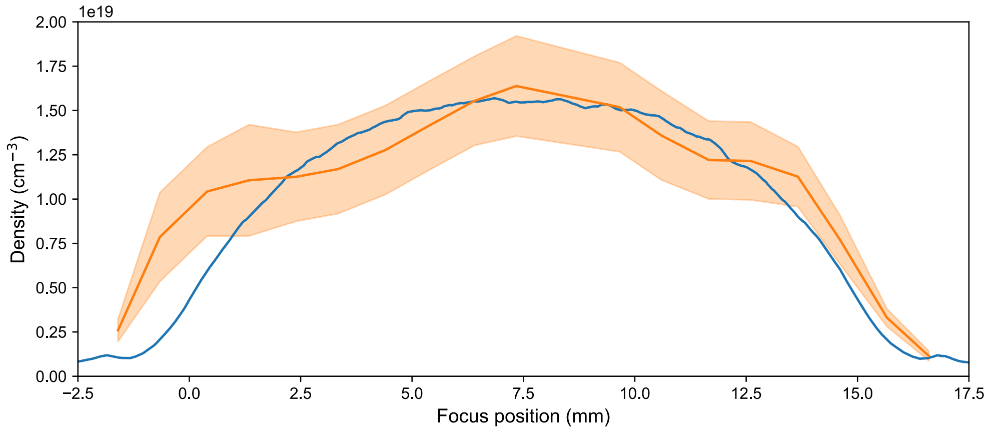

Figure S3: Longitudinal density profile from the fluid simulation (blue curve) and the experiment (orange curve). The colored area indicates the confidence interval of the experimental measurement.

Channel generation. The plasma channel was produced using a 27 fs laser pulse with a 1.46 mJ energy, focused over a line by an axiparabola ([1, 2]). The resulting laser intensity along the focal line was $\approx 5 \times$

10^{15} W cm^{-2}, which leads to the ionization of the gas around the laser axis through optical field ionisation (OFI). The channel formation was studied with numerical simulations using the experimental parameters. The modelling of the plasma channel formation process included: (a) the optical propagation of the axiparabola beam, (b) OFI and resulting electron heating, (c) plasma expansion. These there steps are illustrated in Fig. S4(a-c). Note that in Fig. 2b of the paper, as in Fig. S4c, we plot the distribution of $n_\mathrm{p} = \rho_\mathrm{p}/A_\mathrm{H}$, where ρ_p is the mass density of the media and $A_\mathrm{H} \simeq 1$ is the atomic weight of Hydrogen. It differs from the experiment where we measured the density of free electrons.

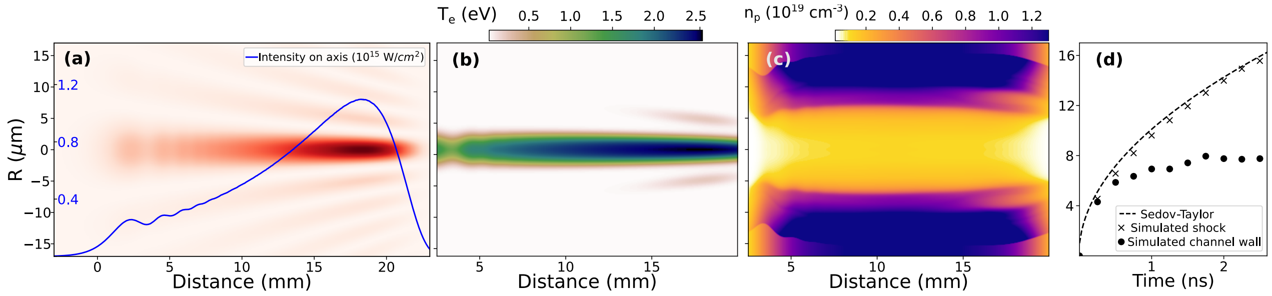

Figure S4: (a) Distribution of laser flux logarithm $\ln\left(\int E^2 dt\right)$ (red colors, normalized), and on-axis intensity (blue curve) along the focal line. (b) Electron temperature provided by OFI process. (c) Distribution of electrons $n_p = \rho_p/A_\mathrm{H}$ after 2 ns of expansion. (d) Propagation of the radial shock front in simulation (crosses) and theoretical model (dashed line), and of the channel wall (circles).

In the simulations the propagation of the axiparabola beam is modelled in the axisymmetric cylindrical geometry, which does not take into account the beam asymmetries produced by the off-axis axiparabola in the experiment. Nevertheless, the simulation describes well the details of the formation of the plasma channel. The simulated results have also been verified, against the Sedov-Taylor expansion theoretical model, which defines the shock motion as (using the same expression as in Eq.(2) of [3]):

$$r_s(t) = (\gamma_\mathrm{ai} + 1)^{1/2}\,(Z_\mathrm{i} E_\mathrm{k}/M_\mathrm{i})^{1/4}\big(r_0(t + t_0)\big)^{1/2} \tag{1}$$

where adiabatic index $\gamma_\mathrm{ai} = 5/3$, E_k is the initial kinetic energy of electrons, Z_i and M_i are the ion charge and mass and r_0 and t_0 are the constants corresponding to the initial size radius of heated electrons and time off-set of the expansion. For the actual interaction parameters, Eq. (1) is shown in Fig. S4(d) (dashed line) along with the shock front position extracted from the simulation (crosses), and the models show a good agreement. One may also note, that the channel formation dynamics does not follow exactly the one of the shock-wave, and in Fig. S4(d) we can see that at $t \approx 1-2$ ns the channel expansion ceases, while the shock front still propagates. This is caused by the plasma cooling – during the first ≈ 1 ns of expansion hot electrons flow away from the axis, and the plasma temperature on-axis falls down to ≈ 0.2 eV, thus stopping its expansion.

Longitudinal and transverse plasma expansion. Since the electron temperature produced by OFI is nearly constant along the focal line, plasma expansion is mainly radial. At the same time, in the density transition injection case, with the hydrodynamic shock placed perpendicular to the axis, the OFI-heated plasma of this small scale density shock contains longitudinal pressure gradients. The expansion rate of a shock (temperature or pressure spike) with a typical scale-length L_grad is determined by the plasma temperature, and occurs on a time-scale $\sim L_\mathrm{grad}/\langle v_s\rangle$, with $\langle v_s\rangle$ being the time-averaged ion-acoustic velocity of a collisionless hydrogen plasma $v_s = \sqrt{kT_\mathrm{e}/m_\mathrm{p}}$. In our experiment, the scale lengths of the channel and the shock are ~ 20 μm and ~ 200 μm respectively, and the initial electron temperature produced by OFI is $1.5-2.5$ eV. From the spatial scale discrepancy of the longitudinal and transverse features follows that the corresponding expansions would also proceed on very different times. Moreover, as the plasma temperature rapidly falls during the radial expansion (on a time of ~ 1 ns), the resulting longitudinal expansion turns out to be negligible.

The above conclusion is confirmed by the simulation of the channel formation with a shock located at $z = 5.1$ mm. The plasma density map presented in Fig. S5a, does not show any visible signs of a longitudinal increase of the shock size. The isolated shock profile calculated by the subtraction of the same case but without a shock is presented in Fig. S5b. A minor deviation between initial shock profile and one developed after 2 ns corresponds to the increase of the shock RMS size by 3 μm, and has no effect on the laser-plasma acceleration performance.

Waveguide optimization. In the experiment the waveguide optimization was performed by using, the imaging of Thomson scattering from the main laser, the density profile measurements, the data from the electron spectrometer, and the measurements of the transmitted laser modes. For one of these studies, we varied the

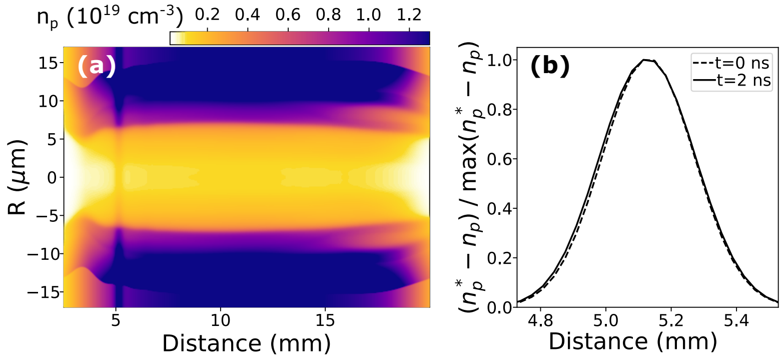

Figure S5: (a) Simulated distribution of plasma density $n_{\mathrm{p}} = \rho_{\mathrm{p}}/A_{\mathrm{H}}$ after 2 ns of expansion, with the shock. (b) Simulated shock profile at different times.

delay between the generation and the accelerating laser beams in the region from 0.5 ns to 4 ns. In this scan we observed an optimum in terms of the accelerated charge and maximum electron energies for the delays between 1.5 and 2.5 ns, while laser guiding continue to be effective for longer delays.

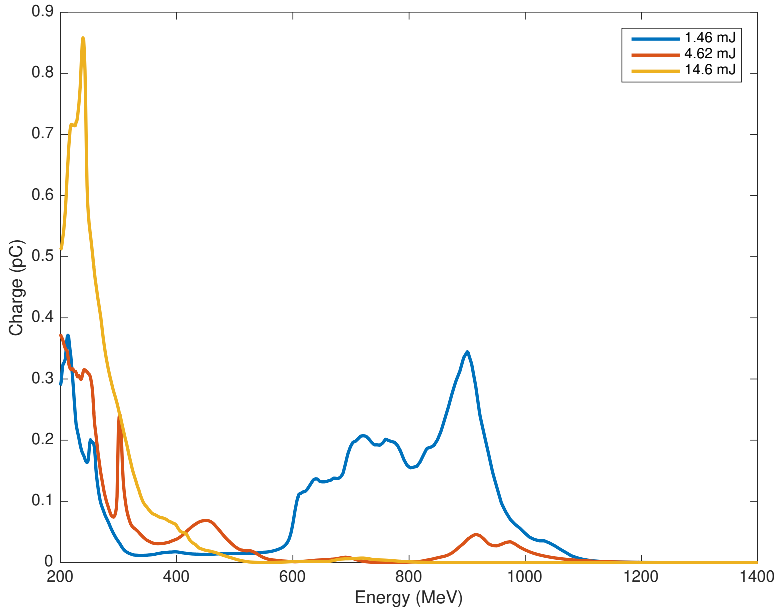

Figure S6: Electron spectra measured for three different energies of the laser pulse P2. Each spectrum is averaged over 5 consecutive shots.

Another optimisation was realized by varying the energy of the channeling laser, using a half wave-plate coupled with a polarizer. We have found, that for the laser energies beyond a few milli-Joules, electron acceleration performance drastically degrades, while the driver laser is still well-guided. This effect is demonstrated in Fig. S6, where the optimal energy of ~ 1.5 mJ was found. Most likely the performance degradation results from the fact that for higher laser energies, OFI heating occurs not only on the laser axis but also at the fringes, which results into additional shock waves converging towards the axis and perturbing the plasma density profile at channel's bottom.

Correlations between the quality of guiding and the electron beam properties. In the experiment with optimal parameters, about 30% of shots still failed to produce high-energy electron bunches with a substantial charge. Figure S7 shows the focal spots at the exit of the waveguide and the corresponding electron spectra, for a series of 10 consecutive shots, without any exclusion. For these shots, electrons are trapped in the accelerator via ionization injection, and therefore have continuous energy distributions. They were acquired without the spectrometer collimator, leading to a larger systematic error, which is estimated here at 125 MeV at 1 GeV. In order to facilitate comparison, the same normalization is used for all shots.

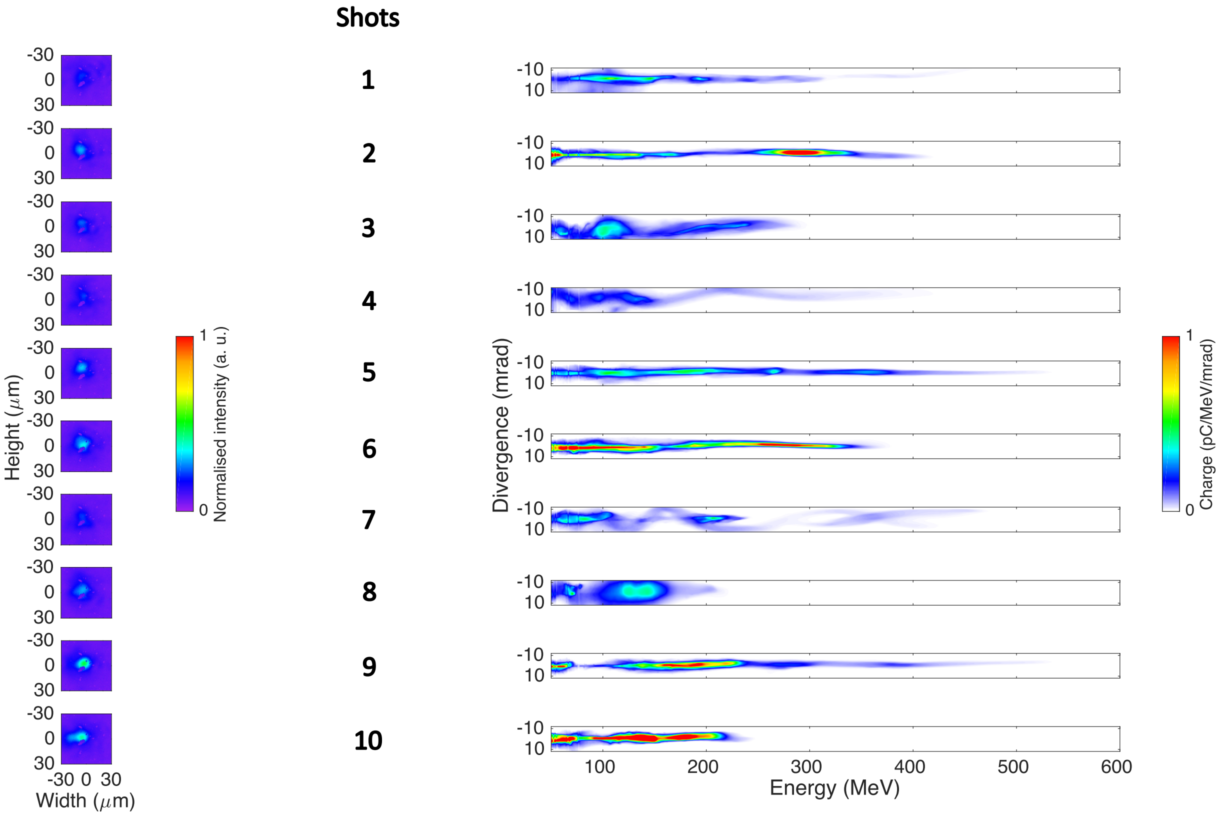

Figure S7: Focal spots and electron spectra from 10 consecutive shots.

In Fig. S7, a clear correlation between an efficient guiding and the charge at high energy is observed. In the shots 2, 5, 6 and 9 the beam was well guided and electrons reach GeV-level energies. In contrast, an inefficient guiding (shots 1, 3, 4, 7 and 8) results in a very low or even zero charge at high energies. Figure S7 also shows that when the laser is not correctly guided, electron spectra exhibit phase oscillations, i.e. correlations between the emission angle and the energy. These oscillations are consistent with a laser beam that would enter the waveguide with an angle, perform oscillations in the waveguide and lead to off-axis injections. These observations suggest that the accelerator stability is mainly limited by the coupling of the laser into the waveguide. The lack of stability of this coupling can be traced back to the 2-3 μrad (RMS) pointing fluctuations of the P1 laser beam which are comparable to the transverse size of the plasma channel.

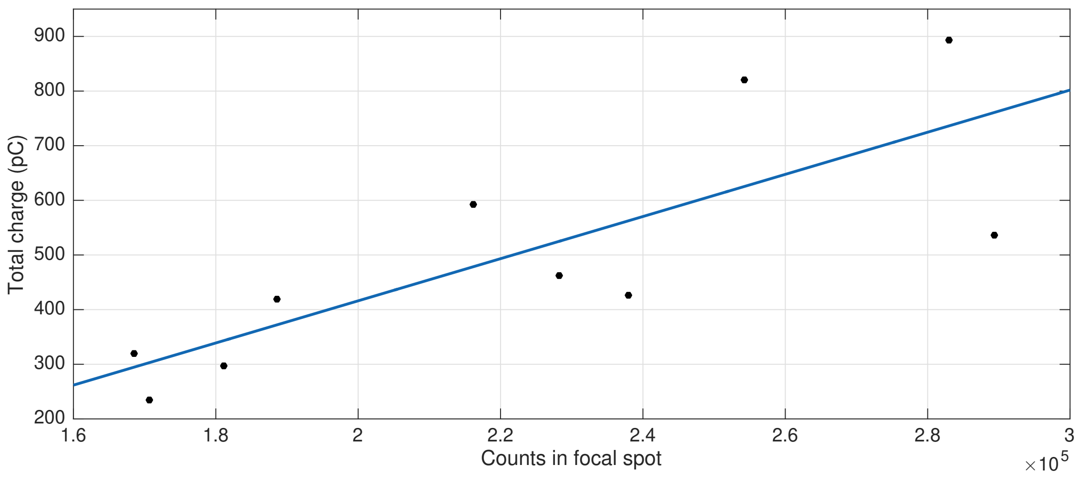

Figure S8: Correlation between the total charge of the electron bunch and the counts in the focal spot area.

As ionization injection leads to the continuous trapping of electrons into the wakefield, the total charge of the bunch is a good figure of merit to quantify the quality of guiding, as it is less sensitive to beamloading than the maximum energy. The beamloading can explain the case of shot 10, where a well-guided focal spot

produces no charge at high energies, no significant oscillations, but a total charge being the highest of the series. The corresponding scaling is illustrated in Fig. S8 which depicts the total measured charge as a function of the counts in the central focal spot[1] for every shot represented in Fig. S7. The high coefficient of correlation $r = 0.79$ confirms the link between the electron injection and the quality of guiding.

References

[1] S. Smartsev, C. Caizergues, K. Oubrerie, J. Gautier, J.-P. Goddet, A. Tafzi, K. Ta Phuoc, V. Malka, and C. Thaury. Axiparabola: a long-focal-depth, high-resolution mirror for broadband high-intensity lasers. *Optics Letter*, 44(14):3414–3417, 2019.

[2] K. Oubrerie, I. A Andriyash, R. Lahaye, S. Smartsev, V. Malka, and C. Thaury. Axiparabola: a new tool for high-intensity optics. *Journal of Optics*, 24(4):045503, 2022.

[3] R. J. Shalloo, C. Arran, L. Corner, J. Holloway, J. Jonnerby, R. Walczak, H. M. Milchberg, and S. M. Hooker. Hydrodynamic optical-field-ionized plasma channels. *Physical Review E*, 97:053203, 2018.

[1] The counts are integrated over a disk whose radius corresponds to the first zero radius of the focal spot at the entrance of the waveguide
